# Differences in Population-Based Dietary Intake Estimates Obtained From an Interviewer-Administered and a Self-Administered Web-Based 24-h Recall

**DOI:** 10.3389/fnut.2020.00137

**Published:** 2020-08-27

**Authors:** Didier Brassard, Catherine Laramée, Julie Robitaille, Simone Lemieux, Benoît Lamarche

**Affiliations:** ^1^Centre de Recherche FRQ-S Nutrition, Santé et Société (NUTRISS), Institut sur la Nutrition et les Aliments Fonctionnels (INAF), Université Laval, Quebec City, QC, Canada; ^2^School of Nutrition, Université Laval, Quebec City, QC, Canada

**Keywords:** 24-h recall, web-based, interviewer-administered, survey, underreporting, Quebec, CCHS, PREDISE

## Abstract

Web-based instruments are being increasingly used in nutrition epidemiology and surveillance. However, the extent to which dietary intake estimates derived from web-based 24-h recalls such as the R24W are consistent with data derived from more traditional interviewer-administered 24-h recalls (TRAD) remains uncertain. Our objective was to compare dietary intake estimates obtained using the R24W and a TRAD instrument in population-based samples from the province of Québec in Canada. This comparison of dietary assessment methods was based on data from two sample survey studies in adults (18–65 years). The R24W was used in a sample of 1,147 French-speaking adults from five regions of Québec as part of the PREDISE (*PRÉDicteurs Individuels, Sociaux et Environnementaux*) study. The TRAD was used in a sample of 875 French-speaking adults from the Canadian Community Health Survey 2015 located in the same five regions. Characteristics of both samples were matched through selection and weighting (language, sex, age, region, education, body mass index, weekend day, and season of survey). Mean and usual intake data of each sample were compared. The plausibility of reported energy intakes was compared using predictive equations of the Institute of Medicine. Mean servings/day from the R24W were higher than with TRAD for vegetables and fruit (+11%, *P* = 0.003), grain products (+7%, *P* = 0.06), milk and alternatives (+21%, *P* < 0.001), and meat and alternatives (+18%, *P* = 0.001). Intake of low nutritive value foods was also 28% higher with the R24W than with TRAD (mean difference +164 kcal; 95% CI, 107–222). As a result, total energy intakes from the R24W compared with TRAD were 18% higher in women (mean difference +325 kcal; 95% CI, 243–407) and 15% higher in men (mean difference +361 kcal; 95% CI 232–490). The prevalence of underreporting of energy intakes was 10% lower with the R24W than with TRAD (prevalence ratio 0.90; 95% CI, 0.86–0.94). In conclusion, differences between dietary assessment methods in the context of population-based surveys on nutrition have potentially important consequences on the quality of the data and should be carefully considered in future surveys and surveillance strategies.

## Introduction

Population-based surveys are essential to guiding nutrition-oriented public health policies (1). When collected regularly, nutrition surveys provide valuable data regarding trends in a population's dietary intakes, nutrient intake adequacy, food insecurity, and impact of diet-related policies, which can then inform the development of dietary recommendations (2). In Canada, the most recent methodology in national nutrition survey employed face-to-face interviewer-administered 24-h recall assisted by computer (hereafter referred to as TRAD) (3). Dietary intake is assessed using a recall instrument because it is considered less biased than other instruments such as food frequency questionnaires (FFQ) or dietary screeners (4–6). In the case of the FFQ, respondents report the frequency of consumption of a finite list of foods and over periods generally ranging from 1 month to 1 year. Accordingly, intakes assessed using a FFQ depend on one's perceived usual intakes and do not reflect foods actually consumed on a particular day (6). While using a similar approach, a screener focuses on shorter list of foods [e.g., vegetables and whole fruits (6)]. Although the use of a 24-h recall is known to produce higher quality intake data, the training of the interviewers, the impromptu visits to conduct interviews and the review of the 24-h recall coding are technical and monetary barriers to frequent nutrition survey data collection using TRAD.

Technological advances have prompted the development and the broader use of web-based dietary assessment instruments (7–9). Collecting dietary intake data using a self-administered web-based 24-h recall is efficient as it greatly reduces burden on the research team. It also standardizes questions and offers the respondent the ability to complete the 24-h recall at the time of the day and place deemed appropriate (10–12). Food and nutrient intake estimates derived from an interviewer-administered 24-h recall and web-based 24-h recall instruments are generally consistent (13–15). However, data suggest that digital pictures of web-based 24-h recall may attenuate misestimation of portion size compared with food booklet pictures of an interviewer-administered 24-h recall (16). It is stressed that meticulous dietary assessment interviews conducted by expert teams in research settings often may not reflect the methodology employed in a large national survey. For example, interviewers involved in the Canadian Community Health Survey 2015 (CCHS 2015) to conduct the dietary assessment using TRAD received 2.5 days of training, but were not required to have a formal degree in nutrition (3). The consistency of dietary intake data obtained in the CCHS 2015 with a more contemporary dietary assessment methodology is not well-documented to date. Thus, comparing data from a web-based 24-h recall with data from an interviewer-administered 24-h recall as used in the national survey is essential to best inform on future nutrition survey methodology.

Our team has developed and validated a web-based 24-h recall instrument, the R24W (9, 17–19), which we used subsequently to assess the diet of an age- and sex-representative sample of 1147 French-speaking adults from five regions in the Province of Quebec, Canada from 2015 to 2017 as part of the PREDISE (*PRÉDicteurs Individuels, Sociaux et Environnementaux*) study (20, 21). Because CCHS and the PREDISE study were conducted within the same period in similar population-based samples, we have a unique opportunity to compare the consistency of dietary intake data obtained using TRAD and a web-based 24-h recall. The overall aim of the present study was therefore to compare distribution of food and nutrient intake estimates obtained using a TRAD and the R24W in similar population-based samples from the Province of Quebec. The primary outcome of this analysis was the difference between mean food intake estimates (vegetable and fruit, grain products, milk and alternatives, meat and alternatives, and “other foods”) between TRAD and the R24W. As exploratory outcomes, we investigated mean differences for nutrients of public health interest, namely, energy, saturated fats, sodium, sugar, potassium, and fibers. We hypothesized that the dietary assessment method influences dietary intake estimates.

## Methods

### Study Design and Participants

This comparison of dietary assessment methods was based on two population-based surveys in French-speaking adult men and women (18–65 years) living in the Province of Quebec. The present study objectives and methods were pre-specified in contract #18-SSH-LAVAL-5901 with Statistics Canada as part of the standard procedure to be granted access to confidential microdata files of CCHS 2015.

#### The PREDISE Study

Complete methods of the PREDISE study have been published elsewhere (20). Briefly, a multicenter cross-sectional and web-based study was designed to examine the association between individual, social, and environmental factors and adherence to Canadian dietary guidelines. Participants were men and women from five major administrative regions of the Province of Quebec in Canada. To be eligible, participants had to be 18–65 years of age, speak French as primary language at home, have a computer, have access to the Internet, and have a valid email address. Participants were recruited by a survey company using random digit dialing and a quota-based approach for sampling between August 2015 and April 2017. The majority of 24-h recalls (67%) were collected in 2016; 30 and 3% of dietary intake data were collected in 2015 and 2017, respectively. The quotas were based on 30 strata created on the basis of the five administrative regions (*Capitale-Nationale/Chaudière-Appalaches, Estrie, Mauricie, Montréal*, and *Saguenay-Lac-St-Jean*), sex, and three predetermined age groups (18–34, 35–49, and 50–65 years). Each stratum was based on the most recent demographic data from the *Institut de la statistique du Québec* (2013) at the time of recruitment, aiming for a sample size of 1,000 participants. Those who completed all questionnaires were eligible for a draw to win one of the 40 gift cards and two electronic devices. The PREDISE study protocol was approved by the ethics board from each participating institution.

#### The Canadian Community Health Survey 2015

CCHS 2015 is a nationally representative survey of individuals aged 1 year and older living in private dwellings located among 10 Canadian provinces (3). Full-time members of the Canadian Forces, individuals living in the Territories, on reserves, in remote areas, or in institutions were excluded from CCHS 2015. Data were collected evenly throughout the year from January 1st to December 31st, 2015. Respondents from CCHS 2015 were selected to match inclusion criteria of the PREDISE study. To be included, CCHS 2015 respondents had to be located in one of the five administrative regions of the PREDISE study, which are designated as census metropolitan area within CCHS 2015, to be 18–65 years of age and to speak French as the primary language at home. Pregnant and lactating women were excluded. Analyses of CCHS 2015 data were conducted at Université Laval's Research Data Center using the “Master Files” (Version 2) (3). The ethical approval for CCHS 2015 Nutrition conducted by Statistics Canada is based on the authority of the Statistics Act of Canada.

### Dietary Assessment *via* 24-h Recalls

#### The PREDISE Study (R24W)

In PREDISE, participants were invited by email to complete a self-administered web-based 24-h recall, the R24W, on three separate unannounced occasions selected randomly by an in-house computer algorithm during a 21-days period. Details about the development and validation of the R24W have been reported elsewhere (9, 17–19). Briefly, the R24W was inspired by the automated multiple-pass method of the *United States Department of Agriculture* and uses a meal-based approach to begin the recall (9). The R24W automatically generates dietary intakes data of food groups defined according to Canada's Food Guide 2007 (22) and of nutrients using the Canadian Nutrient File 2015 (23). As detailed elsewhere (21), consumption of low nutritive value foods (termed “other foods”) was also examined using the Health Canada Surveillance Tool tier system (24). According to this classification, foods high in saturated fats, sodium, sugars, and/or total fats are flagged when they exceed predetermined thresholds for those nutrients. Pastries, sweets, sugar-sweetened beverages, processed meats, and French fries are some examples of foods included in the low nutritive value food category. Only data from the first R24W were used in the present study to compute mean intakes consistent with the CCHS 2015 methodology (see below). Data from a single 24-h recall, despite a well-characterized within-individual random error, are adequate to provide an estimation of mean usual intakes at the group level (6). However, to compare distribution of dietary intakes, usual intake estimate distributions were computed using the National Cancer Institute (NCI) methods 2.1 (25). The NCI methods use regression calibration to mitigate within-person random error caused by day-to-day variation in intakes, among others, but require the availability of repeated measurements. Only 20% of respondents in CCHS 2015 had completed a second 24-h recall. Data from the second recall in a random sample of 20% of all participants in PREDISE were used when applying the NCI method in this sample to have a consistent proportion of repeated recalls between surveys.

#### The Canadian Community Health Survey 2015 (TRAD)

In CCHS 2015, the first 24-h recall was administered by trained interviewers in person *via* computer-assisted interviews based on the Automated Multiple Pass Method (3). Only data from the first 24-h recall was used to calculate mean intakes. A food booklet was provided to respondents to help in estimating portion sizes of food and beverage in plates, bowls, glasses, and mugs (3). Intake of Canada's Food Guide 2007 food groups was computed. Nutrient intakes were also computed using the Canadian Nutrient File 2015 (23). Intake of “other foods” was examined using the same criteria as in PREDISE (21). Usual dietary intake estimate distributions were also computed using the NCI methods 2.1 and the second TRAD 24-h recall collected in 20% of the sample. The second 24-h recall was collected by phone by trained interviewers.

### Clinical Assessment

In PREDISE, participants who completed all online questionnaires were invited for clinical assessment in the nearest affiliated research center. Height was measured with a height gauge and weight was measured in the fasted state to the nearest 0.1 kg (Tanita, BWB-800S; Arlington Heights, Il). In CCHS 2015, the interviewers measured the height and weight of respondents with a standard scale to the nearest 0.01 kg (LifeSource Scales Model US-321).

### Assessment of Systematic Error

Large-scale, routine assessment of energy expenditure using a gold standard method (i.e., doubly labeled water) is not realistic in the context of population-based surveys. In the absence of such method, the use of predictive equation is considered appropriate to assess the plausibility of energy intake (26). The plausibility of self-reported energy intakes (rEI) was assessed using the method proposed by Huang et al. (27). The same method has previously been used in CCHS 2004 and 2015 (28) and in PREDISE (21). Predicted energy requirements (pER) were calculated using the equations provided by the Institute of Medicine (29). An objective measure of physical activity was lacking in both studies; thus, all respondents were assumed to have a sedentary level of activity. This assumption is reasonable considering that only 15% of Canadian adults met physical activity guidelines when measured using accelerometry in the Canadian Health Measure Survey (30). Participants were classified as underreporters, plausible reporters, or overreporters on the basis of their ratio of rEI:pER ratio. A ratio of 1.00 indicates exact correspondence between both estimates. To account for measurement errors and normal variation in energy expenditure, the confidence limits were calculated using the approach proposed by Garriguet (28). The same cutoffs were used in both PREDISE and CCHS 2015 to get a consistent classification of the plausibility of reported energy intakes. Thus, under- and overreporters had a rEI to pER ratio <0.70 and >1.42, respectively (28).

### Statistical Analyses

The statistical software package SAS was used for analyses (Studio version 3.8 for PREDISE and version 9.4 for CCHS 2015). The statistical software R and the ggplot2 package were used to produce box-and-whisker plots (31).

#### Sample Balancing

Sample balancing according to *a priori* determined sociodemographic characteristics was applied to increase comparability between the CCHS 2015 and PREDISE samples (Supplementary Table 1). These characteristics were sex (men and women), age group (18–34, 35–49, 50–65), administrative region (*Capitale-Nationale/Chaudière-Appalaches, Estrie, Mauricie, Montréal*, and *Saguenay-Lac-St-Jean*), education level (no diploma, high school, CEGEP or trade school, university), body mass index [normal (<25 kg/m^2^), overweight (25–29.9 kg/m^2^), obese (>29.9 kg/m^2^)], season when the dietary assessment was conducted (Jan–Mar, Apr–Jun, Jul–Sept, and Oct–Dec), and the proportion of 24-h recalls on weekdays vs. weekend days (Mon–Thu vs. Fri–Sun). Since person-level sampling weights are required to balance samples, missing data had to be imputed for education level and body mass index category. Missing data regarding education levels (PREDISE *n* = 60; 5% of the sample) and body mass index (PREDISE *n* = 130; 11% of the sample, CCHS *n* = 210; 24% of the sample) were imputed once using the fully conditional specification method. A SAS macro available online was used to automate the iterative sample-balancing process (32). The study-specific corrected sampling weights were calculated by sex and were then used in all subsequent analyses.

#### Variance Estimation

For PREDISE, a variable representing the 30 sampling strata was included within the STRATA statement of SAS' survey-specific procedures for variance estimation owing to the stratified sampling. The Taylor series method was used to compute standard errors and 95% confidence intervals in PROC SURVEYMEANS and in PROC SURVEYFREQ. For CCHS 2015, the 500 bootstrap weights provided by Statistics Canada were used to compute standard errors and 95% confidence intervals via the balanced repeated replication method in PROC SURVEYMEANS and PROC SURVEYFREQ. A domain analysis was performed in both surveys to stratify results according to sex.

#### Usual Intakes (NCI Methods)

For PREDISE and CCHS 2015, covariates of the usual intake models were sequence of recall, age, sex, and weekend day and season of survey. For CCHS 2015, the model for “other foods” intakes yielded very high within:between variance ratio (above 50), indicating that between-individual variance could not be distinguished from within-individual variance. Two outliers who had a mean difference between the second and first 24-h recall >2.5 standard deviations away from the mean of differences were identified (33). Removal of data for these outliers rendered the within:between variance ratio acceptable (i.e., 13.8). Distributions of usual intakes were compared using box-and-whisker plots.

#### Comparisons Between Samples

Differences in dietary intake data between the PREDISE sample estimated using the R24W and the CCHS 2015 sample estimated using TRAD were calculated as absolute difference and percentage difference [(R24W – TRAD)/TRAD × 100]. Hypothesis testing was performed using independent Student's *t*-tests. To perform the *t*-tests, finite population standard deviations were calculated for each variable in each survey. Degrees of freedom for the *t*-tests were 1,117 in PREDISE (*N* minus number of strata) and 500 in CCHS (number of replicate weights).

In PREDISE and CCHS 2015, the plausibility of reported energy intakes was missing in a minority of respondents (*n* = 130 and *n* = 210, respectively), because of missing height and weight data. Therefore, we performed multiple imputation (50 times; PROC MI and PROC MIANALYZE) of missing “reporting status” using the FCS method in both studies. To compare samples, prevalence ratios were calculated to assess the likelihood of underreporting, defined as a binary outcome (underreporting vs. else) using univariable log-binomial regression models.

## Results

### Flow Chart and Participants' Characteristics

**Figure 1:**
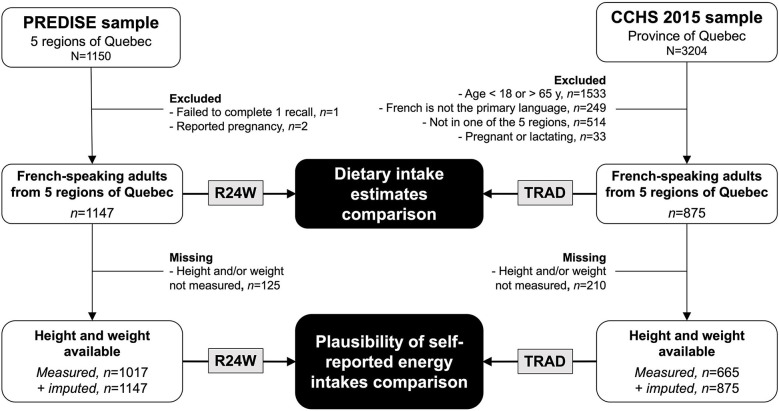
Participant flow chart. CCHS 2015, Canadian Community Health Survey 2015. PREDISE, *PRÉDicteurs Individuels, Sociaux et Environnementaux*.

A total of 1,147 respondents from PREDISE and 875 respondents from CCHS 2015 were included in the present study (Figure 1). Their weighted characteristics after balancing of the two samples are presented in Table 1. Compared with the CCHS 2015 sample, the proportion of respondents with a household income above 90,000 $CAD was 6.2% lower in the PREDISE sample. In CCHS 2015, 96% of interviews were conducted in person, while 4% were conducted partially or completely by telephone. Dietary intake data in PREDISE were collected through the web in all participants.

**Table 1:** Characteristics of the CCHS 2015 and PREDISE (2015–2017) samples from Quebec, Canada.

**Category**	**Level**	**CCHS 2015, *N* = 875**	**PREDISE, *N* = 1147**	**Difference**
Sex^*^	Men	436 (49.8)	571 (49.8)	-
	Women	439 (50.2)	576 (50.2)	-
Age group, years^*^	18–34	312 (35.6)	408 (35.6)	-
	35–49	258 (29.5)	338 (29.5)	-
	50–65	305 (34.9)	400 (34.9)	-
Region^*^	*Capitale-Nationale*-*Chaudière-Appalaches*	332 (37.9)^F^	435 (37.9)	-
	*Estrie*	84 (9.6)	110 (9.6)	-
	*Mauricie*	75 (8.6)^F^	99 (8.6)	-
	*Montréal*	303 (34.6)^F^	397 (34.6)	-
	*Saguenay-Lac-Saint-Jean*	81 (9.3)^F^	107 (9.3)	-
BMI group^*^	Normal (<25 kg/m^2^)	354 (40.4)	464 (40.4)	-
	Overweight (25–29.9 kg/m^2^)	315 (36.0)	413 (36.0)	-
	Obese (>29.9 kg/m^2^)	206 (23.6)	270 (23.5)	−0.1
Education^*^	Less than high school	96 (11.0)	126 (11.0)	-
	High school	175 (20.0)	229 (20.0)	-
	CEGEP or trade school	289 (33.0)	379 (33.0)	-
	University	315 (36.0)	413 (36.0)	-
Household income, CAD$	<30,000	127 (14.6)	197 (20.0)	+5.4
	30,000 to <60,000	249 (28.5)	282 (28.7)	+0.2
	60,000 to <90,000	168 (19.2)	194 (19.7)	+0.5
	≥90,000	330 (37.8)	310 (31.6)	−6.2
Currently smoking	No	714 (81.9)	959 (83.6)	1.7
	Yes	158 (18.1)	188 (16.4)	−1.7
Taking dietary supplement	No	623 (71.2)	771 (67.2)	−4.0
	Yes	252 (28.8)	376 (32.8)	+4.0
Season of 24-h recall^*^	Winter	219 (25.0)	287 (25.0)	-
	Spring	219 (25.0)	287 (25.0)	-
	Summer	219 (25.0)	287 (25.0)	-
	Fall	219 (25.0)	287 (25.0)	-
Day of completion of the 24-h recall^*^	Weekday	499 (57.0)	654 (57.0)	-
	Weekend	376 (43.0)	493 (43.0)	-

*Values are n (percent)*.

* *Variables that were used for the sample-balancing process. Differences between samples were not applicable in such cases*.

*^F^Indicates unreliable estimates as per Statistics Canada's standards (coefficient of variation > 33.3%)*.

### Total Foods Reported and Food Intake Estimate Comparison

**Figure 2:**
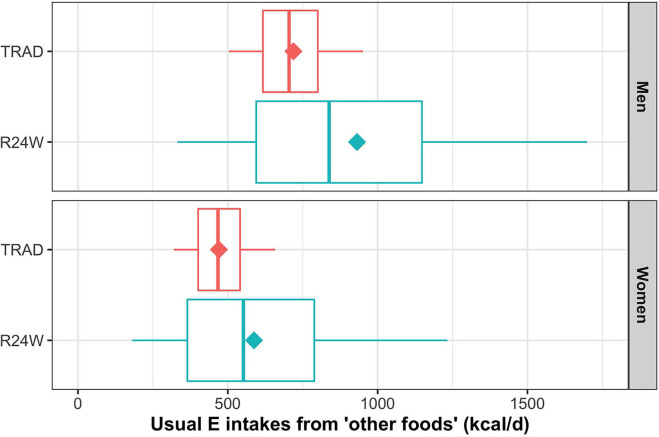
Box-and-whisker plot of energy intakes from “other foods” (kcal) by dietary assessment instrument and sex. The NCI methods 2.1 (amount model) were used to compute usual intake distribution. Boxes represent quantiles 0.25, 0.50, and 0.75. Lower and upper whiskers are respectively quantiles 0.05 and 0.95. Diamonds are mean. Data were adjusted for age, sex, administrative region, education level, body mass index, weekend, and season through the sample balancing process using sex-specific sampling weights. E, energy.

**Figure 3:**
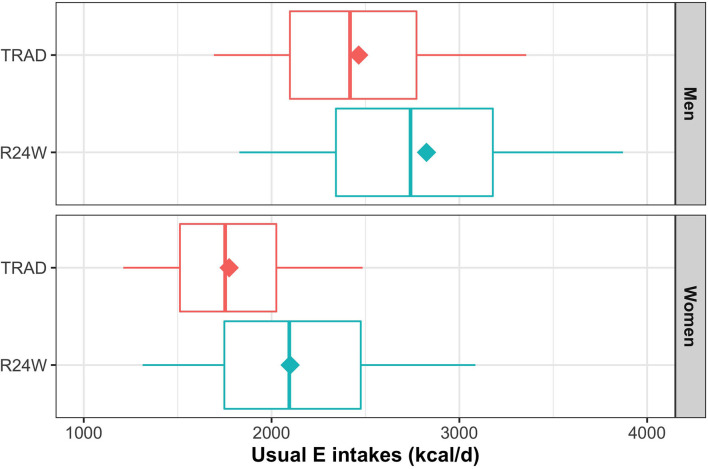
Box-and-whisker plot for self-reported energy intakes by dietary assessment instrument and sex. The NCI methods 2.1 (amount model) were used to compute usual intakes distribution. Boxes represent quantiles 0.25, 0.50, and 0.75. Lower and upper whiskers are respectively quantiles 0.05 and 0.95. Diamonds are mean. Data were adjusted for age, sex, administrative region, education level, body mass index, weekend, and season through the sample balancing process using sex-specific sampling weights. E, energy.

On average, respondents reported consuming a mean of 18.8 food items each day with the R24W and 16.2 items with TRAD (Supplementary Table 2). Self-reported intake of Canada's Food Guide 2007 food groups assessed using the R24W were all higher than values obtained with TRAD (Table 2). The difference in reported intake between the R24W and TRAD was largest for the milk and alternatives food group (+21%; *P* < 0.001) and for the meat and alternatives food group (+18%; *P* = 0.001). Self-reported intake of “other foods” was 28% higher (*P* < 0.001) with the R24W than with TRAD (Figure 2; Supplementary Table 2). As a result, mean energy intakes measured by the R24W compared with TRAD were 15% higher in men (mean difference 361 kcal; 95% CI, 232–490; *P* < 0.001) and 18% higher in women (mean difference 325 kcal; 95% CI, 243–407; *P* < 0.001; Figure 3; Supplementary Table 2). The full distribution of usual energy intakes obtained using data from the R24W compared with TRAD shifted toward higher intakes in men and women (Supplementary Figure 1).

**Table 2:** Mean Canada's Food Guide-2007 servings using an interview-administered 24-h recall (TRAD) and a web-based 24-h recall (R24W) in population-based samples of French-speaking adults from Quebec, Canada.

	**Variable**	**Servings/day**			Percentage difference^*^	***P*-value^†^**
		**TRAD**	**R24W**	**Absolute difference (95% CI)**		
**All**						
	Vegetables and fruit	4.9 (0.2)	5.4 (0.1)	0.5 (0.2,0.9)	11%	0.003
	Grain products	5.4 (0.2)	5.8 (0.1)	0.4 (−0.0,0.7)	7%	0.06
	Milk and alternatives	1.7 (0.2)	2.1 (0.1)	0.4 (0.2,0.5)	21%	<0.001
	Meat and alternatives	2.1 (0.2)	2.5 (0.1)	0.4 (0.2,0.6)	18%	0.001
**Men**						
	Vegetables and fruit	5.2 (0.3)	5.4 (0.2)	0.3 (−0.2,0.7)	5%	0.24
	Grain products	6.4 (0.4)	6.7 (0.2)	0.3 (−0.2,0.7)	4%	0.29
	Milk and alternatives	1.8 (0.2)	2.2 (0.1)	0.4 (0.2,0.6)	22%	<0.001
	Meat and alternatives	2.6 (0.3)	3.0 (0.2)	0.4 (0.0,0.8)	15%	0.04
**Women**						
	Vegetables and fruit	4.6 (0.5)	5.4 (0.2)	0.8 (0.4,1.2)	17%	<0.001
	Grain products	4.4 (0.2)	4.9 (0.2)	0.4 (0.1,0.8)	10%	0.005
	Milk and alternatives	1.7 (0.2)	2.0 (0.1)	0.3 (0.2,0.5)	21%	<0.001
	Meat and alternatives	1.7 (0.1)	2.1 (0.1)	0.4 (0.2,0.5)	23%	<0.001

*TRAD and R24W values are mean (SE) intake in servings/day according to Canada's Food Guide 2007 serving sizes. TRAD estimates were obtained using data from the Canadian Community Health Survey (2015). R24W estimates were obtained using data from the PREDISE study (2015–2017). Estimates were balanced using a priori survey- and sex-specific sampling weights accounting for sex, age, region, education level, body mass index, season, and weekend. Sample sizes are 875, 436, and 439 for all, men, and women, respectively (TRAD); 1147, 571, and 576 for all, men, and women respectively (R24W)*.

* *% difference calculated as (R24W – TRAD)/TRAD × 100*.

*P-values (independent Student's t-tests, two-sided) show compatibility of the difference with the null hypothesis of no difference in mean intakes between TRAD and R24W*.

### Nutrient Intake Estimates Comparison

Self-reported intakes of saturated fats, total sugars, sodium, potassium, and fibers were all higher with the R24W than with TRAD (range in difference 10–26%; Table 3). These differences between instruments were observed within each sex (Table 3). Usual intake distributions of saturated fats, total sugars, and sodium are presented in Supplementary Figure 2.

**Table 3:** Mean absolute nutrient intakes using an interview-administered 24-h recall (TRAD) and a web-based 24-h recall (R24W) in population-based samples of French-speaking adults from Quebec, Canada.

**Sex**	**Variable**	**TRAD**	**R24W**	**Absolute difference (95% CI)**	Percentage difference^*^	***P*-value^†^**
**All**						
	Saturated fats, g	27 (3)	33 (1)	6 (4,8)	23%	<0.001
	Total sugars, g	102 (7)	118 (3)	16 (9,24)	16%	<0.001
	Sodium, mg	3,165 (166)	3,470 (83)	305 (116,494)	10%	0.002
	Potassium, mg	2,958 (73)	3,332 (62)	375 (245,505)	13%	<0.001
	Fibers, g	18 (1)	23 (0)	5 (4,6)	26%	<0.001
**Men**						
	Saturated fats, g	30 (3)	37 (1)	7 (4,9)	23%	<0.001
	Total sugars, g	117 (10)	137 (6)	20 (10,30)	17%	<0.001
	Sodium, mg	3,674 (184)	3,940 (124)	266 (2,529)	7%	0.05
	Potassium, mg	3,308 (179)	3,634 (84)	326 (162,491)	10%	<0.001
	Fibers, g	19 (1)	24 (1)	5 (4,7)	27%	<0.001
**Women**						
	Saturated fats, g	23 (2)	28 (1)	5 (4,7)	23%	<0.001
	Total sugars, g	86 (5)	99 (3)	13 (7,19)	15%	<0.001
	Sodium, mg	2,660 (172)	3,004 (102)	344 (184,505)	13%	<0.001
	Potassium, mg	2,610 (135)	3,034 (81)	424 (296,552)	16%	<0.001
	Fibers, g	17 (2)	21 (1)	4 (3,5)	24%	<0.001

*TRAD and R24W values are mean (SE) intake per day. TRAD estimates were obtained using data from the Canadian Community Health Survey (2015). R24W estimates were obtained using data from the PREDISE study (2015–2017). Estimates were balanced using a priori survey- and sex-specific sampling weights accounting for sex, age, region, education level, body mass index, season, and weekend. Sample sizes are 875, 436, and 439 for all, men, and women, respectively (TRAD); 1147, 571, and 576 for all, men, and women respectively (R24W)*.

* *% difference calculated as (R24W – TRAD) / TRAD × 100*.

† *P-values (independent Student's t-tests, two-sided) show compatibility of the difference with the null hypothesis of no difference in mean intakes between TRAD and R24W*.

### Plausibility of Self-Reported Energy Intakes Comparison

**Figure 4:**
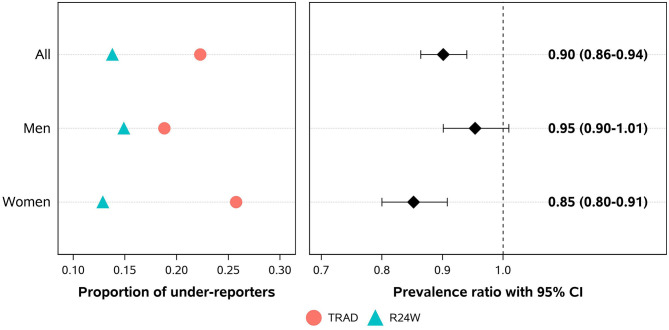
Proportions and prevalence ratios of total energy intake underreporting using a self-administered web-based 24-h recall (R24W) compared with an interviewer-administered 24-h recall (TRAD) in comparable population-based samples. Data were adjusted for age, sex, administrative region, education level, body mass index, weekend, and season of survey through the sample balancing process using sex-specific sampling weights.

The estimated prevalence of underreporting was higher with TRAD (22.3%, 95% CI 11.8–32.7%) than with R24W (13.8%, 95% CI 10.2–17.4%, Figure 4). Thus, the prevalence ratio of underreporting of total energy intakes was lower using the R24W than when using TRAD (prevalence ratio, 0.90; 95% CI 0.86–0.94), and particularly among women (prevalence ratio, 0.85; 95% CI 0.80–0.91; Figure 4). Inversely, the prevalence of overreporting was higher with R24W (19.3%; 95% CI, 16.0–22.5%) than with TRAD (11.4%; 95% CI, 7.5–15.3%; Supplementary Table 3).

## Discussion

Using population-based sample survey studies, we have compared dietary intake estimates obtained using a self-administered web-based 24-h recall (the R24W) to an interviewer-administered 24-h recall (TRAD). Our comparison revealed that intake estimates of most food groups and nutrients were higher when obtained from the R24W than from TRAD. In particular, data revealed that self-reported intake of “other foods,” a category that includes mostly low nutritive value foods, is higher with the R24W than with TRAD. As a result, total energy intake measured by R24W was less likely to be underreported than energy intake measured by TRAD, even more so among women than among men. These data suggest that the use of a web-based 24-h dietary recall may attenuate the prevalence of energy underreporting in the context of nutrition surveys, a major critic of current dietary assessment methods relying on self-reported intakes.

In the present study, French-speaking adults from Québec reported consuming more food items with R24W than with TRAD, which has contributed to the systematically higher food and nutrient intake estimates with the R24W. Of note, the underestimation of low nutritive value foods on TRAD compared with the R24W accounts for ~50% of the difference in total energy intakes between the two instruments. Thus, the greater prevalence of underreporting on TRAD is partially explained by specific underestimation of low nutritive value foods intake. Social desirability bias is known to influence self-reported dietary intakes (34). Indeed, self-reporting dietary intakes in person may be intimidating when conducted in-person (35) and less so when using a web interface, therefore yielding intake data that are less likely to be biased and therefore more likely to be accurate. Differences observed in food and nutrient intakes between the two instruments may be due, in part, to differences in portion size estimation. Indeed, close to 1,500 digital pictures of serving sizes are available within the R24W (9), while a total of 30 pictures are available in the entire CCHS' food booklet (3). Kirkpatrick et al. (16) compared dietary intake data obtained from a web-based 24-h recall (the ASA24) with data from an interviewer-administered 24-h recall in a non-population-based sample. They provided evidence that portion size estimation may be more accurate using numerous digital pictures. In addition, the standardized interface within web-based applications may limit variability of the 24-h recall interviews attributable to the interviewers *per se* (36). In sum, assessing dietary intake with a web-based 24-h recall is likely to yield different dietary intake estimates compared with an interviewer-administered 24-h recall, possibly through a lower degree of desirability bias, standardized questions, and more accurate portion size estimation.

Andreeva et al. compared dietary intake data obtained using a web-based 24-h recall (the NutriNet-Santé web dietary record) to data from a nationally representative sample obtained using interviewer-administered 24-h recall (37). Samples were matched for several sociodemographic characteristics, thus allowing comparison of dietary intake data obtained by the two instruments. Mean dietary intake estimates obtained through the web-based and interview-administered 24-h recalls were similar for most foods and macronutrients. However, intake estimates of sweetened non-alcoholic beverages were lower with the web-based 24-h recall than with the interviewer-administered 24-h recall (−48% in men and −44% in women). Similar proportions of respondents (12–15%) were identified as potential underreporters when using either the interviewer-administered or web-based 24-h recalls. These data are at odds with data from the present study, where important differences were noted between the two measurement approaches. In the study by Andreeva et al., respondents knew beforehand when the web-based 24-h recall would have to be completed, thus potentially triggering reactivity bias (38), which was not the case in the present study. Plus, the interview-administered 24-h recalls were conducted by phone in Andreeva et al., while the majority of respondents in CCHS 2015 (TRAD) were interviewed in person. In-person interviews may have increased the risk of selective underreporting due to more impactful social desirability biases compared with a phone interview (35). This, in turn, may explain the more pronounced differences between web-based and interview-based instruments when in-person interviews are conducted in population-based surveys. Studies to date that compared telephone with in-person interviews have reported inconsistent results regarding the potential impact of social desirability biases on total energy intakes (39–41). Additional studies are therefore needed to determine the extent to which the use of web-based dietary assessment tools alleviates some of the social desirability biases seen with interviewer-based methods.

Other studies have employed a more controlled design using repeated measurements and directly compared dietary intakes obtained using interviewer-administered and web-based 24-h recalls. In a study comparing an interviewer-based 24-h recall and the web-based ASA24 relative to known dietary intakes, Kirkpatrick et al. found that while dietary intakes assessed using both instruments were consistent, the interviewer-administered 24-h recall performed somewhat better than the web-based instrument relative to true intakes (14). Thompson et al. (15) found that intake of energy and the majority of foods and nutrient measured using the web-based ASA24 were similar to values obtained with interviewer-administered 24-h recall. In the same study, only fruits (lower intake using the ASA24) and meat (higher intake using the ASAS24) were considered non-equivalent to data from the interviewer-administered 24-h recall. Timon et al. (42) found that energy, saturated fats, sugars, potassium and sodium were all lower using the web-based Foodbook24 instrument, compared with an interviewer-administered 24-h recall. Intake of most food groups were also lower using the FoodBook24 compared with the interviewer-administered 24-h recall (42). Overall, within-individual comparisons of intake data obtained using an interviewer-administered 24-h recall and a web-based 24-h recall revealed either a similar performance of both instruments (15) or a better performance of the interviewer-administered 24-h recall (14, 42). These observations are also inconsistent with findings from the present study. It is stressed that these studies, which are experimental in nature, have been conducted by expert teams, thus potentially yielding data of greater quality overall compared to data obtained by non-expert interviewers in national surveys. It is also possible that each web-based instrument may have intrinsic features that differentially influence the quality of the measurement. In that regard, Timon et al. (43) reviewed numerous web-based 24-h recall instruments, highlighting that these instruments widely differ in terms of the number of foods considered, the portion size estimation methods and the use of prompts. These differences between instruments must be kept in mind when comparing results among different studies. Finally, only the comparison of self-reported dietary intakes against gold standard method such as doubly labeled water can determine which instrument produces the most accurate estimates. Metabolomics may also help improve current dietary intake assessment based on self-reported approaches (44).

This study has several strengths, including the carefully matched samples between CCHS 2015 (TRAD) and PREDISE (R24W), temporal proximity of both study, reasonable sample size, and the use of the same reference food and nutrient tables to assess food group and nutrient intakes. In this context, we believe that differences between dietary intake estimates assessed using a web-based and an interview-based 24-h recall are plausibly related to intrinsic characteristics of each instrument. These characteristics include, among others, the mode of administration (i.e., web vs. interviewer-administered) and the portion size estimation support (i.e., digital pictures vs. food booklet). Limitations must also be discussed. First, this comparison of dietary assessment methodology is opportunistic and, consequently, the comparison of dietary intake estimates may also reflect true differences in intake between samples and different degree of selection bias. In particular, the PREDISE sample (R24W) included slightly fewer smokers and a higher proportion of dietary supplement users, suggesting a tendency toward healthier lifestyle habits. Second, missing data for height and weight led to a greater proportion of participants with “imputed” energy intake reporting status in the CCHS 2015 sample and this may have also biased our assessment of the plausibility of self-reported energy intakes in this sample. In particular, missing data regarding reporting status may be associated with body weight, potentially contributing to bias if the imputation model does not fully capture the relationship between body weight and reporting status based on the data available. The wide 95% confidence intervals in CCHS 2015 are indeed compatible with a low to moderate prevalence of underreporting (12–33%). Third, the lack of an objective measure of physical activity limited our ability to assess the proportion of plausible reporters of energy intake, but this was the case in both samples.

In conclusion, this comparison of dietary intake estimates obtained in matched population-based samples of adults from Québec revealed systematically higher intake of foods and nutrient when using a web-based 24-h recall compared to using a traditional, interview-administered 24-h recall. These differences, which are not trivial, are plausibly related to the dietary assessment methodology considering the similar population examined and the temporal proximity of both dietary assessments. Such differences between instruments need to be considered along with other characteristics such as cost, efficiency of administration, and burden of data management when determining which dietary assessment instruments is best suited for use in surveillance and population-based surveys.

## Data Availability Statement

For the PREDISE study, the raw data supporting the conclusions of this article will be made available by the authors, without undue reservation. For the Canadian Community Health Survey 2015—Nutrition (CCHS), only the summary data supporting the conclusions of this article will be made available by the authors, without undue reservation. The raw CCHS data used in this article is not readily available because the confidential microdata files can only be accessed through Research Data Centers in Canada. Instructions to access the raw data can be found at https://www.statcan.gc.ca/eng/microdata/data-centres/access.

## Ethics Statement

The studies involving human participants were reviewed and approved by 1.1) PREDISE STUDY (online study protocol): Laval University Ethics Committee (# 2014–271); 1.2) PREDISE STUDY (clinical assessment study protocol): ethics board of each participating institution (Laval University, #2014–271; Montreal Clinical Research Institute, #2015–02; Université du Québec à Trois-Rivières, #15–209- 07.13; Center Hospitalier Universitaire de Sherbrooke and ECOGENE-21 Biocluster, #MP-31-2015-997); 2) CANADIAN COMMUNITY HEALTH SURVEY (CCHS): The ethical approval for CCHS 2015 Nutrition conducted by Statistics Canada is based on the authority of the Statistics Act of Canada. The patients/participants provided their written informed consent to participate in this study.

## Author Contributions

DB and BL designed the study. DB performed statistical analyses and wrote the first draft of the manuscript. SL, BL, JR, and CL have developed and validated the R24W. All authors critically reviewed the manuscript, provided final approval of the submitted manuscript, had full access to all of the data in the study, take responsibility for the integrity of the data, the accuracy of the data in the analysis, affirm that the article is an honest, accurate, and transparent account of the study being reported and that no important aspects of the study have been omitted.

## Disclosure

DB has received a speaking honorarium from the Dairy Farmers of Canada in 2018. DB is the recipient of a doctoral training award from the *Fonds de recherche du Québec–Santé*. DB has been a casual employee at the Office of Nutrition Policy and Promotion (Health Canada) in 2019 and 2020. SL, BL, JR, and CL have developed and validated the R24W, which is currently used for research purposes exclusively.

## Conflict of Interest

The authors declare that the research was conducted in the absence of any commercial or financial relationships that could be construed as a potential conflict of interest.
