# Supplementary material for: Differences in Population-Based Dietary Intake Estimates Obtained From an Interviewer-Administered and a Self-Administered Web-Based 24-h Recall
Source: Front Nutr. 2020 Aug 27;7:137. doi: 10.3389/fnut.2020.00137 (PMC7481356; doi:10.3389/fnut.2020.00137)
Supplement: Supplementary file 1 [file Data_Sheet_1.PDF]

Supplementary Material

1 Supplementary Methods

Supplementary Table 1. Control proportions used to balance samples

Characteristics	Proportions, %	
**Sex***		
Men	49.8	
Women	50.2	
**Age group, y***		
18-34	35.6	
35-49	29.5	
50-65	34.9	
**Administrative region / Census metropolitan area***		
Estrie	9.6	
Saguenay-Lac-Saint-Jean	9.3	
Capitale-Nationale / Chaudière-Appalaches	37.9	
Montréal	34.6	
Mauricie	8.6	
**Education level †**		
Less than high school	11.0	
High school	20.0	
CEGEP or trade school	33.0	
University	36.0	
**Body max index ‡**	**Men**	**Women**
Normal (< 25 kg/m^2)	34.1	46.7
Overweight (25-29.9 kg/m^2)	41.1	31.0
Obese (> 29.9 kg/m^2)	24.8	22.3
**Season**		
Winter (Jan-Mar)	25.0	
Spring (Apr-Jun)	25.0	
Summer (Jul-Sept)	25.0	
Autumn (Oct-Dec)	25.0	
**Weekend / weekday proportion**		
Mon-Thu	57.0	
Fri-Sun	43.0	

* Proportions correspond to the population of each administrative region according to the most recent demographic data from the *Institut de la statistique du Québec (2013)* at the time when the PREDISE study was designed.
† Proportions correspond to those of the whole Province of Quebec in CCHS 2015. CEGEP is a pre-university and technical college institution specific to the Quebec educational system.
‡ Proportions correspond to those of the whole Province of Quebec in CCHS 2015 (annual component; Statistics Canada. Table 13-10-0096-01. Health characteristics, annual estimates)

2 Supplementary Tables

Supplementary Table 2. Mean intakes by dietary assessment instruments in population-based samples of French-speaking adults from Quebec, Canada

Sex	Variable	TRAD	R24W	Absolute difference (95%ci)	% difference*	Pvalue†
**All**						
	Energy, kcal	2118 (86)	2460 (50)	343 (245,440)	16%	<.001
	"Other foods", kcal	594 (57)	759 (27)	164 (107,222)	28%	<.001
	Number of food items reported	16.2 (0.4)	18.8 (0.3)	2.7 (2.1,3.3)	17%	<.001
	Energy per food item, kcal/item	139 (4)	145 (3)	6.1 (-1.9,14.1)	4%	0.14
**Men**						
	Energy, kcal	2464 (113)	2825 (73)	361 (232,490)	15%	<.001
	"Other foods", kcal	719 (71)	931 (43)	212 (134,290)	30%	<.001
	Number of food items reported	16.0 (0.6)	18.2 (0.3)	2.2 (1.5,2.9)	14%	<.001
	Energy per food item, kcal/item	164 (7)	171 (5)	7.1 (-4.9,19.1)	4%	0.25
**Women**						
	Energy, kcal	1774 (68)	2099 (53)	325 (243,407)	18%	<.001
	"Other foods", kcal	470 (56)	587 (24)	117 (62.9,171)	25%	<.001
	Number of food items reported	16.3 (0.4)	19.4 (0.5)	3.1 (2.4,3.9)	19%	<.001
	Energy per food item, kcal/item	113 (4)	118 (2)	5.1 (-0.1,10.3)	5%	0.06

TRAD and R24W values are mean (SE) intake per day. TRAD estimates were obtained using data from the Canadian Community Health Survey (2015). R24W estimates were obtained using data from the PREDISE study (2015-17). Estimates were balanced using a priori survey- and sex-specific sampling weights accounting for sex, age, region, education level, body mass index, season and weekend. Sample sizes are 875, 436, 439 for all, men and women respectively (TRAD); 1147, 571, 576 for all, men and women respectively (R24W).
TRAD, interviewer-administered 24-hour recall; R24W, self-administered web-based 24-hour recall.
* % difference calculated as (R24W - TRAD) / TRAD × 100.
† P values (independent Student's t tests, two-sided) show compatibility of the difference with the null hypothesis of no difference in mean intakes between TRAD and R24W

Supplementary Table 3. Plausibility of self-reported energy intakes by dietary assessment instruments in population-based samples of French-speaking adults from Quebec, Canada

		TRAD		R24W	
**Sex**	**Plausibility of self-reported energy intake**	**n, weighted**	**Proportion (95%CI)**	**n, weighted**	**Proportion (95%CI)**
**All**					
	Under-reporter	195	22.3 (11.8,32.7)	158	13.8 (10.2,17.4)
	Plausible reporter	580	66.3 (56.8,75.8)	768	66.9 (62.6,71.2)
	Over-reporter	100	11.4 (7.5,15.3)	221	19.3 (16.0,22.5)
**Men**					
	Under-reporter	82	18.7 (3.9,33.6) [F]	85	14.8 (10.9,18.8)
	Plausible reporter	311	71.3 (54.3,88.2)	366	64.2 (58.2,70.1)
	Over-reporter	44	10.0 (3.7,16.4)	120	21.0 (15.8,26.2)
**Women**					
	Under-reporter	113	25.8 (16.2,35.3)	74	12.8 (6.8,18.7)
	Plausible reporter	270	61.4 (52.7,70.1)	401	69.7 (63.5,75.8)
	Over-reporter	56	12.8 (6.6,19.0)	101	17.6 (13.8,21.3)

Under- and over-reporters had a ratio of reported energy intakes on the first 24-h recall to predicted energy requirements < 0.70 and > 1.42, respectively (1). Missing values were imputed 50 times using FCS method. TRAD estimates were obtained using data from the Canadian Community Health Survey (2015). R24W estimates were obtained using data from the PREDISE study (2015-17). Estimates were balanced using a priori survey- and sex-specific sampling weights accounting for sex, age, region, education level, body mass index, season and weekend. Sample sizes are 875, 436, 439 for all, men and women respectively (TRAD); 1147, 571, 576 for all, men and women respectively (R24W).

TRAD, interviewer-administered 24-hour recall; R24W, self-administered web-based 24-hour recall.

[F] Indicate unreliable estimate as per Statistics Canada's standards (coefficient of variation > 33.3%)

3 Supplementary Figures

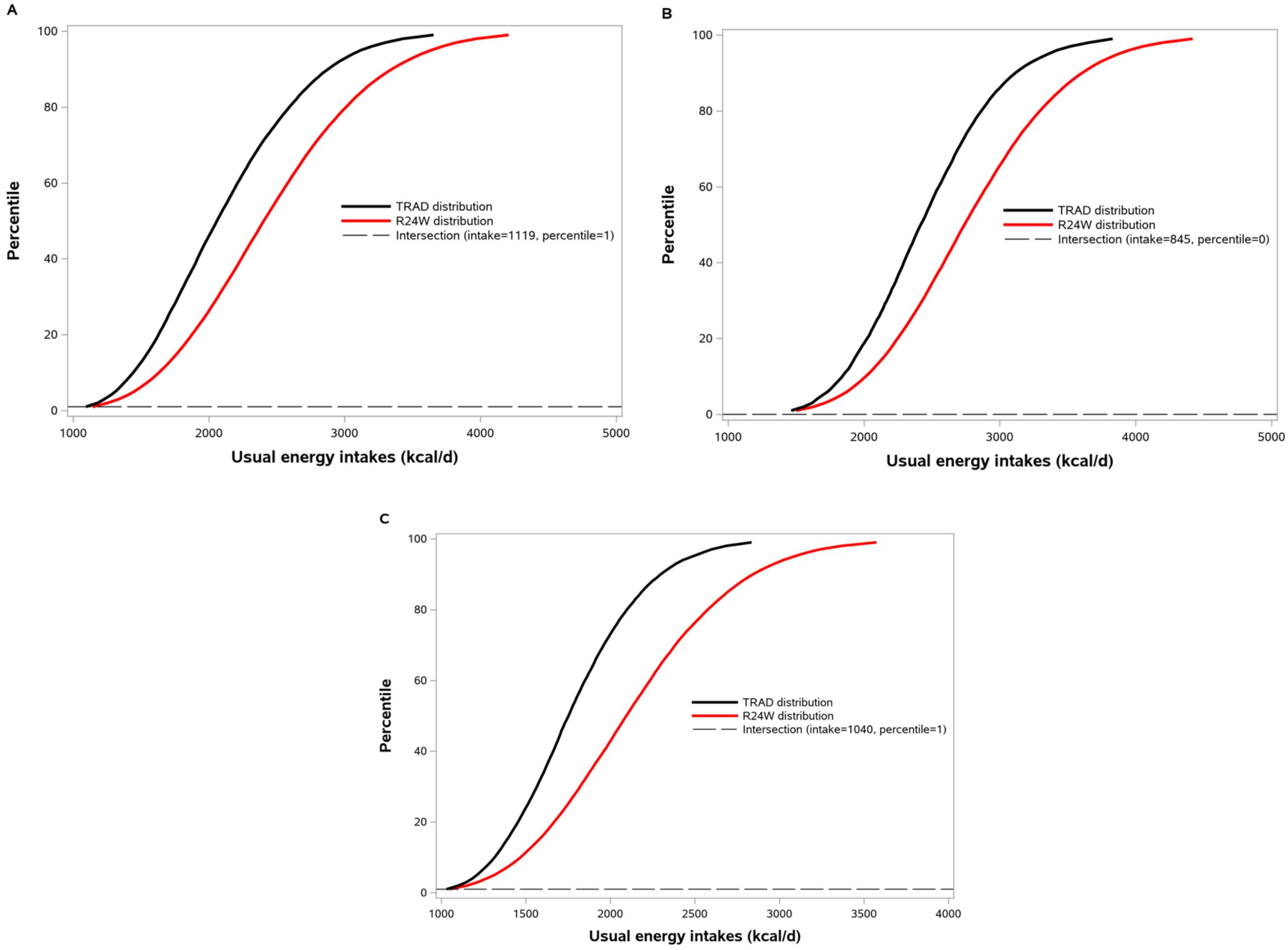

Supplementary Figure 1. Usual energy intakes distribution (kcal/d) in A) all respondents, B) men and C) women. Curves are crossing at the percentile value indicated by the dashed line, if any. The National Cancer Institute methods 2.1 (amount model) were used to compute usual intakes distribution with covariates sequence of recall, weekend indicator, age and sex. TRAD, interviewer-administered 24-hour recall; R24W, self-administered web-based 24-hour recall.

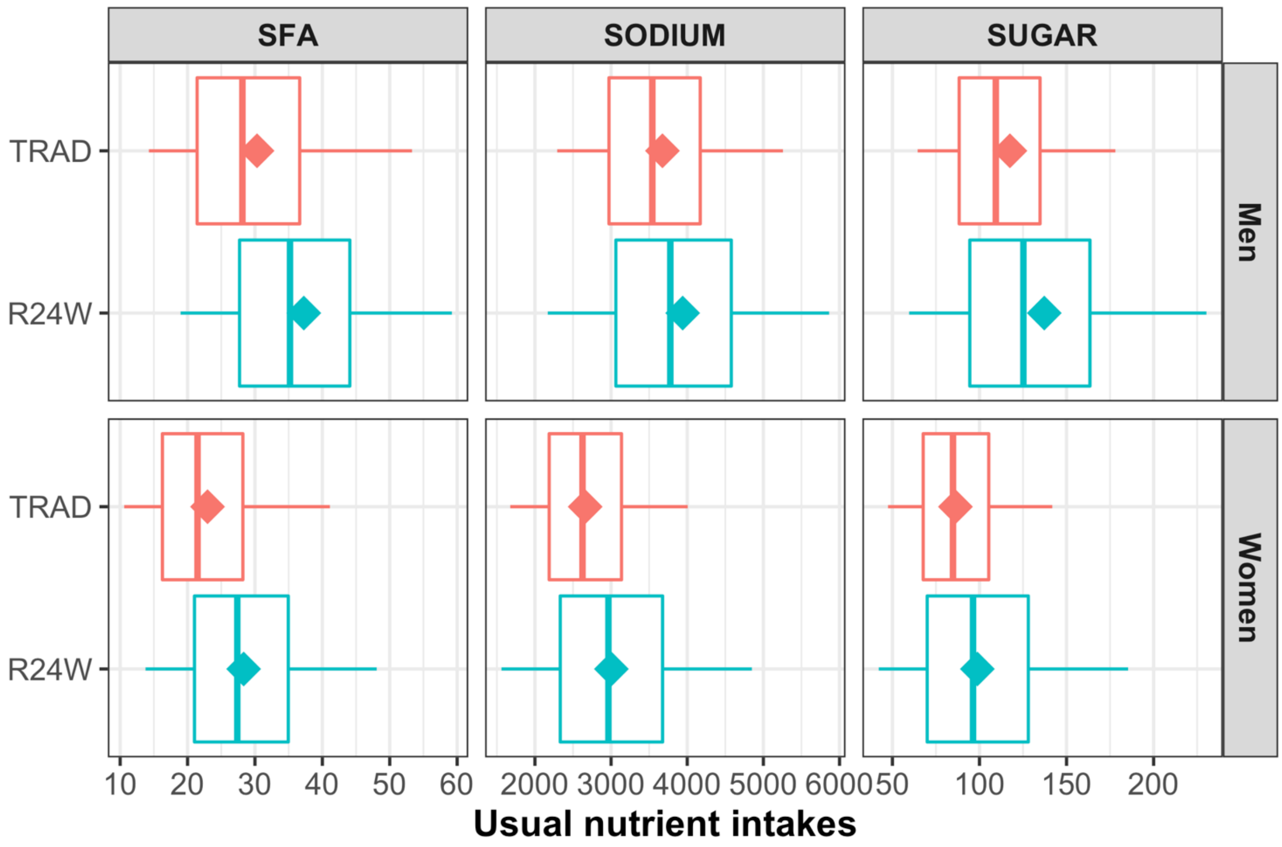

Supplementary Figure 2. Box-and-Whisker plot for intakes of SFA (g/d), sodium (mg/d) and total sugars (g/d) by dietary assessment instrument and sex. The NCI methods 2.1 (amount model) were used to compute usual intakes distribution. Boxes represent quantiles 0.25, 0.50, 0.75. Lower and upper whiskers are respectively quantiles 0.05 and 0.95. Diamonds are mean. Data were adjusted for age, sex, administrative region, education level, body mass index, weekend and season through the sample balancing process using sex-specific sampling weights. R24W, self-administered web-based 24-hour recall; TRAD, interviewer-administered 24-hour recall; SFA, saturated fats.

4 Supplementary References

1. Garriguet D. Accounting for misreporting when comparing energy intake across time in Canada. Health Rep. 2018;29(5):3-12.
